# Multidrug-Resistant *Listeria* Species Shows Abundance in Environmental Waters of a Key District Municipality in South Africa

**DOI:** 10.3390/ijerph18020481

**Published:** 2021-01-08

**Authors:** Liyabona Mpondo, Kingsley Ehi Ebomah, Anthony Ifeanyi Okoh

**Affiliations:** 1 SAMRC Microbial Water Quality Monitoring Centre, University of Fort Hare, Alice 5700, South Africa; kebomah@ufh.ac.za (K.E.E.); aokoh@ufh.ac.za (A.I.O.); 2 Applied and Environmental Microbiology Research Group (AEMREG), Department of Biochemistry and Microbiology, University of Fort Hare, Alice 5700, South Africa

**Keywords:** antimicrobial-resistant gene, *L. monocytogenes*, *Listeria*, multidrug-resistance, public health

## Abstract

The prevalence of bacteria with multidrug-resistance (MDR) is a significant threat to public health globally. *Listeria* spp. are naturally ubiquitous, with *L. monocytogenes* particularly being ranked as important foodborne disease-causing microorganisms. This study aimed to evaluate the incidence and determine the antimicrobial resistance (AMR) profiles of multidrug-resistant *Listeria* spp. (MDRL) isolated from different environmental samples (river and irrigation water) in the Sarah Baartman District Municipality (SBDM), Eastern Cape Province (ECP), South Africa. Molecular identification and characterization were carried out using polymerase chain reaction (PCR) and isolates that exhibited phenotypic resistance were further screened for relevant antimicrobial-resistant genes (ARGs). Findings revealed a total of 124 presumptive *Listeria* isolates; 69 were molecularly confirmed *Listeria* species. Out of the confirmed species, 41 isolates (59%) were classified as *L. monocytogenes* while 9 (13%) were classified as *L. welshimeri*. All *Listeria* spp. exhibited phenotypic resistance against ampicillin, penicillin, and trimethoprim-sulphamethoxazole and further screening revealed ARGs in the following proportions: *sulI* (71%), *bla_TEM_* (66%), *tetA* (63%), and *bla_CIT_* (33%). Results confirmed the occurrence of ARGs among *Listeria* inhabiting surface waters of ECP. The present study indicates that the river water samples collected from SBDM are highly contaminated with MDRL, hence, constituting a potential health risk.

## 1. Introduction

Genus *Listeria* is made up of a group of small rod-shaped, non-spore-forming, facultative anaerobic microorganisms that belong to the family *Listeriaceae*. These bacterial species are motile and capable of thriving at low temperatures and in harsh conditions [1,2]. The genus *Listeria* contains 20 major species including *L. welshimeri*, *L. grayi*, *L. marthii*, *L. seeligeri*, *L. ivanovii*, *L. innocua*, and *L. monocytogenes* and these microbes are present within various environmental niches including soil, vegetables, water, wastewater, and animal feces [3]. Many studies have investigated the occurrence of *Listeria* species in different environmental sources such as soil [4,5,6], while other studies reported on farm samples [7], livestock along with ready-to-eat (RTE) food products [8,9,10,11]. Additionally, some other studies have investigated the prevalence of *Listeria* in wastewater treatment plants (WWTPs) suggesting that these pathogens can thrive even after chlorination [12,13,14,15]. Among the common *Listeria* spp., *L. ivanovii* and *L. monocytogenes* are both considered as relevant animal and human pathogens. However, *L. monocytogenes* is regarded as the third most common foodborne pathogen causing infections in individuals with higher risk, for instance, elderly and immune-compromised people, as well as pregnant women [16]. These infections can lead to a potentially critical and fatal illness known as listeriosis, with extreme cases bringing about symptoms such as meningitis and septicemia in addition to abortion and/or miscarriage in pregnant women [17].

The World Health Organization (WHO) ranked listeriosis among the deadliest foodborne diseases with a 20% to 30% mortality rate [18]. *L. monocytogenes* is a transitory resident of the intestinal tract in humans, with about 10% of the world’s population reported to be carriers of the microorganisms without experiencing any apparent symptoms [19,20]. According to a report by Jemmi and Stephan [21], listeriosis was responsible for a high rate of hospitalization in the United States of America (USA), with post-2009 reports from Europe indicating similar hospitalization rates compared to those reported by the aforementioned study. Furthermore, *Listeria* spp. harboring antibiotic resistance genes (ARGs) have been detected in agricultural samples [22] and the emergence of antibiotic-resistant *L. monocytogenes* (ARLM) has also been reported subsequently [17,23,24].

Although multidrug-resistant *Listeria* spp. (MDRL) have rarely been reported, antibiotic-resistant *Listeria* (ARL) was first reported from a French patient diagnosed with meningoencephalitis in 1988. A recent study disclosed isolates exhibiting phenotypic resistance against streptomycin, chloramphenicol, tetracycline, and erythromycin [16]. Other studies have reported ARL exhibiting resistance against antimicrobials such as aminoglycosides, beta-lactams, chloramphenicol, and also sulphonamides [25,26]. Thus, ARL has also become an increasing One Health concern that heightens the antibiotic resistance challenges faced globally, further undermining progress in healthcare, food production, and life expectancy posing health risks to not only humans but also animals and the environment [27].

In 2011, the Centers for Disease Control and Prevention (CDC) reported an outbreak of listeriosis that was associated with contaminated cantaloupe melons in Colorado, USA which led to 147 cases, out of which 142 were hospitalized, causing 33 fatalities [28]. Recently, CDC reported a multistate outbreak of *L. monocytogenes* infection in the Republic of Korea. The outbreak was linked to commercially bought enoki mushrooms and was responsible for 36 reported cases, 31 hospitalizations, and 4 fatalities [29]. A report by the National Institute of Communicable Diseases stated that in 2018, the South African government reported an outbreak of about 1053 listeriosis cases that was responsible for more than 200 deaths nationwide in which 10 of those fatalities were reported to be from the Eastern Cape Province [30]. Polony, a ready-to-eat (RTE) product, was implicated as the source of the bacterium by whole-genome sequencing (WGS) [30,31]. Human health is linked with the health of animals and the shared environment through the One Health Approach [32]. Therefore, the presence of *L. monocytogenes* in water sources, particularly those used for irrigation and consumption, may lead to the carry-over of potential pathogens from the water source to fresh produce. Upon consumption of contaminated food or drinking water, infectious doses of the bacterium may manifest in either febrile listerial gastroenteritis (non-invasive listeriosis) or invasive listeriosis associated with the aforementioned symptoms [17]. Presently, there is a scarcity of data on the incidence and antibiogram fingerprints of *Listeria* spp. recovered from the aquatic milieu in South Africa. This is one of the few studies within the Eastern Cape Province (ECP) that aimed to evaluate the occurrence, as well as delineate the antibiogram profiles of *Listeria* spp. isolates recovered from river water and irrigation water sources collected from the Sarah Baartman District Municipality (SBDM) in South Africa.

## 2. Materials and Methods

### 2.1. Description of the Study Area and Sample Collection

This study was conducted in the SBDM (geographical coordinates: 33°57′00″ S; 25°36′00″ E) situated in the ECP, South Africa. The sampling sites were the Great Fish River and the Bloukrans River. These rivers flow south and are tributaries of the Kowie River situated near Grahamstown. The Bloukrans River serves different purposes such as irrigation water for nearby farms and also has other functions for livestock farms. The river also serves as a receiving watershed for final effluent discharge from the district’s WWTP. Water samples were collected downstream from the Bloukrans River and farm samples (specifically irrigation water) from some farms in the SBDM. Six samples were collected on a once-off regime in the month of September. Water samples were collected accordingly in duplicates by means of sterile bottles (1-L) and conveyed in an ice-box to the laboratory for bacteriological analysis in no more than six hours of collection.

### 2.2. Processing of Samples

For bacteriological analysis, the standard filtration method was followed as described by Olaniran et al. [15] with modification. The water samples (100 mL) were filtered via membrane filter papers (0.45-μm pore size) (Merck, South Africa) with the aid of a vacuum pump. For enumeration of the *Listeria* spp., each membrane filter was aseptically picked with sterilized forceps and transferred onto the surface of Chromogenic *Listeria* agar (ISO) Base (Oxoid Limited, Basingstoke, UK) mixed with one vial of OCLA (ISO) selective supplement and OCLA (ISO) differential supplement (Oxoid Ltd., Basingstoke, UK). This followed the standards as described previously by Ottaviani and Agosti (ALOA) in ISO 11290–1:1997 used for enumerating *Listeria* spp. including *L. monocytogenes* [33]. The media plates were incubated for 24 h at 37 °C. After the incubation, blue/green colonies with no halo were considered as unconfirmed *Listeria* spp. on the ISO Base. The data were converted to log_10_ CFU per 100 mL [34].

### 2.3. Isolation of Listeria spp. by Enrichment

Due to the slight turbidity of the river and irrigation water samples collected, enrichment was carried out to increase the detection of *Listeria* spp. by inserting the membrane filter into 90 mL of Tryptic Soy Broth (TSB) and afterward incubated aerobically at 37 °C for 16–18 h (Laboratorios CONDA, Madrid, Spain). Following incubation, the second enrichment was done by aseptically transferring 1 mL of the enriched TSB suspension onto 9 mL of Fraser Broth (Oxoid Ltd., Basingstoke, UK), vortexed, and then incubated at 37 °C for a period of 24 h [34]. From the second enriched suspension, 0.1 mL aliquot of the broth was spread onto Chromogenic *Listeria* agar (LCA) (Oxoid Ltd., UK), thereafter supplemented using OCLA (ISO) selective supplement with OCLA (ISO) differential supplement, following the standards as defined by Ottaviani and Agosti (ALOA) in ISO 11290–1:1997 and incubated aerobically for 24 h at 37 °C. After incubation, presumptive *Listeria* isolates (showing blue/green colonies either with or without halos) were subsequently purified onto nutrient agar (NA) (Oxoid Ltd., Basingstoke, UK) and incubation conditions of 37 °C for a period of 24 h were followed. Pure colonies were further inoculated in sterile nutrient broth (NB) following an incubation period of 18–24 h at 37 °C. The overnight culture was stored in 25% glycerol stock and kept in a freezer at −80 °C for further analysis.

### 2.4. DNA Extraction

The boiling method was employed to extract bacterial DNA following the methods described by Maugeri et al. [35]. The presumptive isolates were resuscitated by inoculation into NB and an incubation period of 18–24 h at 37 °C was followed. A loopful from the NB culture was suspended in 200 µL of sterile distilled water contained in 1.5 mL Eppendorf tubes and vortexed. For 15 min, the suspension was heated at 100 °C in an MS2 Dri-Block DB.2A instrument (Techne, Marshalltown, South Africa) to break down the bacterial cells. After heating, the suspension was then centrifuged (at 10,000 rpm for 10 min) in order to take away the cell debris from the DNA material confined within the supernatant. Thereafter, the lysate supernatant was employed as a DNA template in the polymerase chain reaction (PCR) technique.

### 2.5. Molecular Identification of Presumptive Listeria Isolates

The presumptive *Listeria* isolates were molecularly confirmed using the PCR technique. Table 1 summarizes the set of primers of target *Listeria* species, their respective PCR product sizes, and PCR conditions.

### 2.6. Antimicrobial Susceptibility Test (AST) of the Molecularly Identified Listeria Species

All the identified *Listeria* species were put through AST using 15 panels of antibiotics as described by the Kirby–Bauer disc diffusion technique. The results were interpreted accordingly following the Clinical and Laboratory Standards Institute [38] guidelines. Fifteen test antibiotics (Davies Diagnostics (Pty) Limited, South Africa) were selected across 10 families of antimicrobials: aminoglycosides: gentamicin (10 μg), amikacin (30 μg); beta-lactams: ampicillin (25 μg), oxacillin (1 μg), penicillin G (10 μg); cephems: cephalothin (30 μg); fluoroquinolones: levofloxacin (5 µg), ciprofloxacin (5 μg); quinolones: nalidixic acid (30 μg), sulphonamides: trimetroprim-sulphamethoxazole (25 μg); phenicols: chloramphenicol (30 μg); tetracycline: tetracycline (30 μg); lincosamide: clindamycin (2 μg); macrolides: erythromycin (15 μg) and vancomycin (30 μg). Approximately 100–200 mL of the overnight bacterial broth culture was poured into 5 mL normal saline solution and then standardized using the 0.5 McFarland standard. Subsequently, 100 mL was evenly spread on Mueller–Hinton agar (MHA) (Merck, Modderfontein, South Africa) plates using a sterile glass spreader, and the MHA plates were left to dry a little before being immersed with the aforementioned antibiotic discs, then incubated at 37 °C for 24 h. Afterward, the interpretative zones of diameter for *Staphylococcus* spp. were referred to in this study because the interpretative criteria for *Listeria* spp. are unavailable in the CLSI guidelines [39].

### 2.7. Multiple Antibiotic-Resistance Phenotypes (MARPs) and Multiple Antibiotic-Resistance Index (MARI)

The MARPs pattern of antimicrobial-resistant *Listeria* species (ARLS) was generated based on isolates that exhibited phenotypic antibiotic resistance (AR) to 3 or more antimicrobial agents as described by Krumperman [40], MARI was also determined for each isolate. MARI values were calculated using the mathematical formula:

MAR Index=a/b

where “*a*” denotes the sum of test antibiotics the isolates displayed resistance to; “*b*” denotes the total sum of antimicrobial agents used.

### 2.8. Molecular Characterization of the Relevant Antimicrobial-Resistant Genes

The PCR profiling of antimicrobial resistance (AMR) determinants of ARLS involved the screening of 10 antimicrobial-resistant genes (ARGs) encoding for relevant β-lactamases following the method previously described by Dallenne et al. [41]. Table 2 summarizes the primers used and amplification conditions for the detection of extended-spectrum beta-lactamases (ESBL) ARGs. The β-lactamases assay comprised of one singleplex PCR for the detection of cefotaximase-Munich (CTX-M) group 8/25 and three multiplex PCR assay, which include multiplex I PCR for detection of Temoneira (TEM), sulphadryl variable (SHV) and oxacillinase (OXA) β-lactamase; multiplex II PCR is for the detection of plasmid-mediated AmpC β-lactamase types including the forkhead-box (FOX) group, Citron Rho-Interacting Serine/Threonine Kinase and the chromosomal EBC family; multiplex III PCR for the detection of carbapenemase including *Klebsiella pnuemoniae* carbapenemase (KPC), Verona integon-encoded metallo-beta-lactamase (VIM) and active-on-imipenem (IMI) genes. A total of 14 ARGs were screened using the PCR technique. Table 3 summarizes the list of primers for the screening of relevant ARGs.

## 3. Results

### 3.1. Enumeration and Distribution of Presumptive Listeria spp. in Water Samples

The results showed that the standard plate count for *Listeria* spp. obtained from the river samples collected from Bloukrans River access point one (BRT) and Bloukrans River access point two (BRD) ranged from 3.49–3.88 log_10_ CFU/100mL. The standard plate count from Duncan Farm irrigation-water (DFW) samples ranged from 0–3.68 log_10_ CFU/100mL. Therefore, the dissemination of presumptive *Listeria* spp. was higher for the BRD sampling site in the SBDM, as compared to the other sampling sites.

### 3.2. Molecular Confirmation and Characterization of the Recovered Listeria Isolates

A total number of 124 unconfirmed *Listeria* isolates were recovered from the water samples analyzed. The results revealed that 55.7% (69/124) harbored the *prs* gene for the confirmation of *Listeria* spp. (Supplementary Figure S1). Further characterization revealed 59% (41/69) of the confirmed *Listeria* isolates were confirmed as *L. monocytogenes* (Supplementary Figure S2) and 13% (9/69) were confirmed to be *L. welshimeri* (Supplementary Figure S3).

### 3.3. Antimicrobial Susceptibility Patterns (ASP) of the Confirmed Listeria spp.

All confirmed *Listeria* spp. were subjected to antibiogram susceptibility testing. The result showed that besides gentamicin and amikacin which had the lowest percentage resistance of 32% and 39%, respectively, the *Listeria* isolates exhibited resistance (100%) against other test antibiotics including penicillin, cephalothin, ciprofloxacin, levofloxacin, trimethoprim-sulphamethoxazole, nalidixic acid, chloramphenicol, tetracycline, erythromycin, vancomycin, clindamycin, and oxacillin. Among the 15 antimicrobial agents, only amikacin and gentamicin had activity against *L. monocytogenes* with a low resistance percentage of 39% and 32%, respectively whereas no *L. welshimeri* isolates exhibited resistance against amikacin. However, *L. welshimeri* exhibited a resistance of 33% against gentamicin.

### 3.4. MAR patterns and MAR Indices

The MAR phenotype patterns and MAR indices of *Listeria* spp. were generated and are summarized in Table 4. Results from the phenotypic resistance profiles of *L. monocytogenes* show four MAR patterns against the antibiotics (Table 4). The four MARP patterns were generated from 13 to 15 antibiotics. The most frequently observed MARP pattern among the *L. monocytogenes* was the AP-PG-KF-CIP-LEV-TS-NA-C-T-ERY-VA-CD-OXA pattern with 41.5% (17/41) of the isolates exhibiting the resistance pattern. The MARI values of all *L. monocytogenes* ranged between 0.87 and 1 (Table 4), which is greater than the 0.2 arbitrary threshold set by Krumperman [40]. MARI values ranging from 0.87 to 0.93 were observed for *L. welshimeri*, which is greater than the 0.2 threshold (Table 4). A total of two MARP patterns were observed for *L. welshimeri* isolates as shown in Table 4; with the most frequently observed MARP pattern being AP-PG-KF-CIP-LEV-TS-NA-C-T-ERY-VA-CD-OXA, observed amongst 66.7% of the isolates.

### 3.5. PCR Profiling of Antimicrobial Resistance Determinants in Listeria spp.

The ARGs screened in this study were chosen due to the high percentage of MAR phenotypes exhibited by the confirmed *Listeria* spp. (Table 4). Isolates were screened for 14 relevant ARGs that belong to five different classes of antibiotics including beta-lactams, phenicols, sulfonamides, tetracyclines, and aminoglycoside. Additionally, the confirmed isolates were screened for 10 relevant ESBL genes. The percentage occurrence and distribution of ARGs harbored by *L. monocytogenes* and *L. welshimeri* are summarized in Table 5.

PCR screening of ARGs of multidrug-resistant *Listeria* spp. (MDRL) revealed that among the ARGs responsible for sulfonamide resistance, only *sulI* was detected in 71% (29/41) of *L. monocytogenes* and 67% (6/9) of *L. welshimeri.* Tetracyclines *tetA* was greatly abundant in *L. welshimeri* by 78% (7/9) and 63% (26/41) of *L. monocyotgenes* harbored *tetA* gene; *tetB*, *tetC*, and *tetM* were not detected amongst the *Listeria* spp. Furthermore, resistance gene *catII* was present in very low levels, with resistance proportions of 7% (3/41) amongst *L. monocytogenes* and no phenicol-resistance gene was detected amongst *L. welshimeri* isolates. Among the screened ESBL determinants, *bla_TEM_* was the most dominant resistance genes with *L. monocyotgenes* isolates displaying great *bla_TEM_* presence of 66% (27/41) compared to *L. welshimeri* with 44% (4/9); *bla_OXA-1_* followed with 17% (7/41) and 22% (2/9) amongst *L. monocytogenes* and *L. welshimeri* isolates, respectively. *L. welshimeri* displayed 33% (3/9) *bla_CIT_* resistance. Supplementary Figures S4 and S5 represent the PCR amplicons of the amplification of *sulI* gene and *tet*A gene, respectively.

## 4. Discussion

The emergence of MDRL poses a serious public health threat to humanity. In this study, our findings provide evidence that the environmental waters of the ECP are probable reservoirs of highly pathogenic *Listeria* spp. harboring ARGs. The standard plate count of the presumptive *Listeria* isolates recovered from the selected sample sites was high and this is in accordance with the findings reported by Bilung et al. [7] and Iwu and Okoh [47]. The South African standard guidelines for domestic and irrigation water use are not specific for bacterial concentration. Based on the potable drinking water and irrigation water quality guidelines set by the South African Department of Water Affairs (DWAF) and World Health Organization (WHO) which is ≤1–1000 CFU/100 mL of fecal coliforms [48,49,50], the standard plate counts observed in this study exceeded the acceptable limits. This may be as a result of fecal contamination due to the function of the river as a receiving watershed or run-off from nearby farms involved in animal husbandry. However, in this study, results showed no occurrence of *Listeria* spp. in the irrigation water samples.

Globally, several studies have also investigated the prevalence of *L. monocytogenes* present in river and irrigation waters. In the United States, *L. monocytogenes* was reported in 43% (604/1405) [51] and 31% (53/170) [52] of water samples collected from river and irrigation waters. Furthermore, the bacterium has also been isolated from water samples collected from the point of sewage discharge (into a river) in Europe [53]. The study of Manjur et al. [54] carried out in Bangladesh also investigated the distribution of *Listeria* spp. confirming the occurrence of the microorganisms in soil and surface water samples. In this study, the percentage occurrences (PO) observed was 55.7% for *Listeria* species and 59.4% for *L. monocytogenes*, while the PO of *L. welshimeri* (13%) was much lower. Similarly, other studies reported *Listeria* spp. with PO of 64% [7] and 33.30% [55], which were recovered from surface water bodies (in Canada) and environmental samples (in Malaysia), respectively. Contrary to our findings, another recent study by Iwu and Okoh [47] reported low PO of *L. monocytogenes* (14%) recovered from irrigation water in ECP, South Africa.

The patterns of phenotypic resistance exhibited by *Listeria* spp. in this study revealed that 86.7% of the isolates displayed MDR against the test antibiotics. In contrast, previous studies reported high susceptibility to penicillin, ampicillin, and tetracycline exhibited by *L. monocytogenes* recovered from pork and slaughterhouses in Brazil [8] and in retail raw food in China [56], respectively. The ineffectiveness of penicillin against *L. monocytogenes* observed in this study agrees with another study of Aras and Ardıç [9], which reported *L. monocytogenes* isolated from turkey and meat samples with resistance percentage of 75% and 66.7% to ampicillin and penicillin, respectively. In contrast, the findings of this study revealed resistance exhibited by *L. monocytogenes* against the beta-lactams ampicillin, penicillin, and cephalothin. Similarly, Olaniran et al. [15] reported resistance against penicillin exhibited by *Listeria* spp. isolated from treated wastewater (WW) final effluents and receiving watersheds.

The *Listeria* spp. in this study exhibited varied susceptibility to amikacin and gentamicin (aminoglycoside class). Likewise, our finding is corroborated by the study of Bilung et al. [7], who revealed effective activity of gentamicin against *Listeria* isolates that were recovered from environmental samples collected from farms in Malaysia. Generally, *Listeria* spp. have been reported to be susceptible to a wide range of antibiotics. However, many studies have reported *Listeria* spp. exhibiting phenotypic resistance against antibiotics used in the treatment of listeriosis such as tetracycline, chloramphenicol, and fluoroquinolones [16,17,57].

The MARP patterns observed in this study revealed *L. monocytogenes* and *L. welshimeri* isolates displaying four and two MARP patterns, respectively. Contrary to studies by Arslan and Özdemir [58] and Conter et al. [59], where *Listeria* spp. recovered from homemade white cheese and also food processing environments, respectively, were reported to exhibit single-drug resistance, MDR against a range of 13 to 15 test antibiotics was observed in the current study. Evaluation of the MAR index of ARLS revealed a MAR index that varied from 0.8 to 1 (Table 4) which is above the arbitrary threshold set by Krumperman [40]. In principle, a MARI value above the set threshold (0.2) suggests that the isolates originate from sources of high antibiotic usage. This may imply that the river waters in this study area are highly contaminated with antibiotics including antibiotic residues (antibiotic resistant bacteria (ARB) and ARGs), or other degradable products. These antibiotics find their way into water bodies via final wastewater effluent discharge, surface runoffs from animal husbandry, agricultural soil upon which manure is used as fertilizer and animal waste from nearby grazing livestock systems [60].

The detection of ARGs harbored by *Listeria* spp. and the increasing rates of *Listeria* infections are becoming major concerns to public health [26]. Furthermore, the selective pressure exerted by the exposure of *Listeria* spp. to antibiotics within an environment niche can increase the transfer of ARGs between *Listeria* spp. and other bacterial species by self-transferable plasmids [16,17]. The confirmed *Listeria* spp. were screened for 24 relevant ARGs, out of which only 7 (29.2%) ARGs (*sulI*, *tetA*, and ESBL) were detected. Our findings revealed that among the ARGs detected, *sulI* (which confers AR against sulphonamides) was the most dominant amongst the *Listeria* spp., particularly *L. monocytogenes*. Another dominant ARG was *tetA*, which confers resistance against tetracycline, followed by *bla_TEM_* among *L. monocytogenes* (66%) and *L. welshimeri* (44%) isolates (Table 5). Similarly, Srinivasan et al. [25] reported a low frequency of ARGs harbored by *L. monocytogenes* recovered from dairy milk farms. Among the ARGs reported included *tetA* (32%), and *sulI* (16%). However, other tetracycline resistance genes screened were not found to be harboured by *L. monocytogenes* [25].

In this study, most of the confirmed *Listeria* spp. that exhibited phenotypic resistance did not harbor ARGs. For instance, all *L. monocytogenes* and *L. welshimeri* exhibited phenotypic resistance against chloramphenicol. However, only 7% of *L. monocytogenes* harbored *catII* gene. A low percentage occurrence against aminoglycosides was observed; however, the screened *Listeria* spp. did not harbor ARG. Other studies of Davis and Jackson [23] and Morvan et al. [61] also reported *Listeria* isolates exhibiting phenotypic resistance against aminoglycosides but did not harbor ARGs.

There is insufficient information concerning the occurrence of ESBLs encoding genes harbored by *Listeria* spp. and this is among the few studies reporting the detection of ESBLs encoding genes harbored by *Listeria* spp. recovered from the aquatic environment in South Africa.

## 5. Conclusions

The findings of this current study revealed the presence of *Listeria* spp. in river water and irrigation water samples. All the confirmed *Listeria* spp. exhibited multidrug resistance (MDR) and harbored more than one ARG. The occurrence of MDR strains of *L. monocytogenes* in the water samples indicates that the bacteria are most likely to be transferred to other ecosystems. The ARGs harbored by the *Listeria* spp. are most likely to be transferred to other bacterial species within the aquatic environment. Therefore, this poses a significant public health risk and environmental crisis that involves the spread of ARB. Therefore, more comprehensive monitoring of the nature of acquisition and spread of ARGs among *Listeria spp.* is needed particularly in the agroecosystem. The final effluent from WWTPs should also be appropriately depurated before discharge into receiving water bodies.

## Figures and Tables

**Table 1:** List of primers of target *Listeria* species, respective amplicon sizes, and the polymerase chain reaction (PCR) cycling conditions.

Species	Gene Target	Primer Sequence (5′ → 3′)	Cycling Conditions	Amplicon Size (bp)	Reference
***Listeria* spp.**	*prs*	F-GCT GAA GAG ATT GCG AAA GAAG R-CAA AGA AAC CTT GGA TTT GCGG	94 °C, 94 °C, 60 °C, 72 °C, 72 °C 3′, 1′, 2′, 1′, 15′	370	[36]
***L. monocytogenes***	*prf A*	F-GATACAGAAACATCGGTTGGC R-GTGTAATCTTGATGCCATCAG	94 °C, 94 °C, 56 °C, 72 °C, 72 °C 5′, 45″, 30″, 1′, 5′	274	[37]
***L. welshimeri***	*iap-F* *LW-R*	F-ATGAATATGAAAAAAGCAAC R-GTGCAGGCGCTGGAGCC	94 °C, 94 °C, 52 °C, 72 °C, 72 °C 4′, 20″, 30″, 1′, 5′	919	[10]

′ represents minutes ″ represents seconds.

**Table 2:** Set of primers and amplification conditions for the detection of extended-spectrum beta-lactamases (ESBL) antimicrobial-resistant genes (ARGs).

Multiplex Name	Primer	Amplicon Size (Base Pair (bp))	Primer Sequence (5′–3′)	Cycling Conditions	Reference
Multiplex I TEM, SHV, and OXA-1-like	*bla_TEM_*,	800	F: ATTTCCGTGTCGCCCTTATTC R: CGTTCATCCATAGTTGCCTGAC F: AGCCGCTTGAGCAAATTAAAC R: ATCCCGCAGATAAATCACCAC F: GGCACCAGATTCAACTTTCAAG R: GACCCCAAGTTTCCTGTAAGTG	94 °C, 94 °C, 60 °C, 72 °C, 72 °C 10′, 40″, 40″, 60″, 7′	[41]
	*bla_SHV_*,	713			
	*bla_OXA-1_*	564			
Multiplex II FOX, CIT, and EBC	*bla_FOX_*	162	F: CTACAGTGCGGGTGGTTT R: CTATTTGCGGCCAGGTGA F: CGAAGAGGCAATGACCAGAC R: ACGGACAGGGTTAGGATAGY ^b^ F: CGGTAAAGCCGATGTTGCG R: AGCCTAACCCCTGATACA	94 °C, 94 °C, 60 °C, 72 °C, 72 °C 10′, 40″, 40″, 60″, 7′	[41]
	*bla_CIT_*	538			
	*bla_EBC_*	683			
Singleplex CTX_M group 8/25	*bla_CTX-M_*	326	F: AACRCRCAGACGCTCTAC ^b^ R: TCGAGCCGGAASGTGTYAT ^b^	94 °C, 94 °C, 60 °C, 72 °C, 72 °C 10′, 40″, 40″, 60″, 7′	[41]
Multiplex III IMP, VIM, and KPC	*bla_IMP_*	139	F: TTGACACTCCATTTACDG ^b^ R: GATYGAGAATTAAGCCACYCT ^b^ F: GATGGTGTTTGGTCGCATA R: CGAATGCGCAGCACCAG F: CATTCAAGGGCTTTCTTGCTGC R: ACGACGGCATAGTCATTTGC	94 °C, 94 °C, 55 °C, 72 °C, 72 °C 10′, 40″, 40″, 60″, 7′	[41]
	*bla_VIM_*	390			
	*bla_KPC_*	538			

′ represents minutes ″ represents seconds ^b^ Y = T or C; R = A or G; S = G or C; D = A or G or T [41].

**Table 3:** List of primers and amplification conditions for the screening of target ARGs.

Antimicrobial Class	Target Genes	Primer Sequence (5′–3′)	Amplicon Size (bp)	Amplification Conditions	References
Sulfonamides	*sul1*	F: TTCGGCATTCTGAATCTCAC R:ATGATCTAACCCTCGGTCTC	822	94 °C, 94 °C, 55 °C, 72 °C, 72 °C 5′, 1′, 1′, 5′, 5′	[42]
	*sulII*	F: CGGCATCGTCAACATAACC R: GTGTGCGGATGAAGTCAG	625	94 °C, 94 °C, 50 °C, 72 °C, 72 °C 5′, 30″, 30″, 1′, 5′	[43]
Beta-lactams	*ampC*	F: TTCTATCAAMACTGGCARCC R:CCYTTTTATGTACCCAYGA	550	94 °C, 94 °C, 60 °C, 72 °C, 72 °C 4′, 45″, 45″, 45″, 7′	[44]
	*Bla_TEM_*	F: TTTCGTGTCGCCCTTATTCC R: CCGGCTCCAGATTTATCAGC	690	94 °C, 94 °C, 60 °C, 72 °C, 72 °C 5′, 30″, 30″, 1.5′, 5′	[45]
Tetracyclines	*tetA*	F: GCTACATCCTGCTTGCCTTC R:CATAGATCGCCGTGAAGAGG	201	94 °C, 94 °C, 55 °C, 72 °C, 72 °C 5′, 1′, 1′, 1′, 5′	[46]
	*tetB*	F: TTGGTTAGGGGCAAGTTTTG R: GTAATGGGCCAATAACACCG	359		[46]
	*tetC*	F: CTTGAGAGCCTTCAACCCAG R: ATGGTCGTCATCTACCTGCC	418		[46]
	*tetM*	F: AGT GGA GCG ATT ACA GAA R:CAT ATG TCC TGG CGT GTC TA	158		[46]
Phenicols	*catII*	F: ACACTTTGCCCTTTATCGTC R:TGAAAGCCATCACATACTGC	543	94 °C, 94 °C, 55 °C, 72 °C, 72 °C 5′, 30″, 30″, 90″, 5′	[44]
Aminoglycosides	*strA*	F: CTTGGTGATAACGGCAATTC R:CCAATCGCAGATAGAAGGC	348	94 °C, 94 °C, 55 °C, 72 °C, 72 °C 4′, 45″, 45″, 45″, 7′	[44]
	*aadA*	F: GTGGATGGCGGCCTGAAGCC R: AATGCCCAGTCGGCAGCG	525		[44]
	*aac(3)- IIa(aacC2) a*	F: CGGAAGGCAATAACGGAG R: TCGAACAGGTAGCACTGAG	428	94 °C, 94 °C, 55 °C, 72 °C, 72 °C 5′, 30″, 30″, 90″, 5′	[42]
	*aph(3)- Ia(aphA1) a*	F: ATGGGCTCGCGATAATGTC R: CTCACCGAGGCAGTTCCAT	600		[42]
	*aph(3)- IIa (aphA2) a*	F: GAACAAGATGGATTGCACGC R:GCTCTTCAGCAATATCACGG	510		[42]

′ represents minutes ″ represents seconds.

**Table 4:** MAR patterns of *L. monocytogenes* isolated from the water samples.

No. of Antibiotics	MARP Patterns	No. of Observed	MARI
	***L. monocytogenes* n = 41**		
13	AP-PG-KF-CIP-LEV-TS-NA-C-T-ERY-VA-CD-OXA	17	0.87
14	AK-AP-PG-KF-CIP-LEV -TS-NA-C-T-ERY-VA-CD-OXA	11	0.93
14	GM-AP-PG-KF-CIP-LEV-TS-NA-C-T-ERY-VA-CD-OXA	8	
15	AK-GM-AP-PG-KF-CIP-LEV-TS-NA-C-T-ER-VA-CD-OXA	5	1
	***L. welshimeri* n = 9**		
13	AP-PG-KF-CIP-LEV-TS-NA-C-T-ERY-VA-CD-OXA	6	0.87
14	GM-AP-PG-KF-CIP-LEV-TS-NA-C-T-ERY-VA-CD-OXA	3	0.93

**Table 5:** Distribution of ARGs in *L. monocytogenes* and *L. welshimeri* isolated from the water samples.

Target Antimicrobials	Antimicrobial-Resistant Genes	*L. monocytogenes*	*L. welshimeri*
Sulfonamides	*sulI*	71% (29)	67% (6)
Beta Lactams	*ampC*	0	0
Tetracyclines	*tetA*	63% (26)	78% (7)
	*tetB*	0	0
	*tetC*	0	0
	*tetM*	0	0
Phenicols	*catII*	7% (3)	0
Aminoglycosides	*strA*	0	0
	*aadA*	0	0
Extended Spectrum Beta-Lactams	*blaTEM*	66% (27)	44% (4)
	*blaSHV*	2% (1)	11% (1)
	*blaOXA-1*	17% (7)	22% (2)
	*blaFOX*	0	0
	*blaDHA*	0	0
	*blaCIT*	2% (1)	33% (3)
	*blaEBC*	0	0
	*blaCTXM-8/25*	0	0

## Data Availability

Data sharing not applicable.

## Supplementary Materials

The following are available online at https://www.mdpi.com/1660-4601/18/2/481/s1, Figure S1. PCR products of the amplification of *prs* gene. Lane 1–8: positive *Listeria* spp. isolates; Lane M: 100 bp molecular weight marker (ThermoFisher^TM^ Scientific, (EU) Lithuana); Lane N: negative PCR control (no DNA). Figure S2. PCR products of the amplification of *prf*A gene. Lane 1–11: positive *L. monocytogenes* isolates; Lane M: 100 bp molecular weight marker (ThermoFisher^TM^ Scientific, (EU) Lithuana); Lane N: negative PCR control (no DNA). Figure S3. PCR products of the amplification of 16S rRNA gene. Lane 1–9: positive *L. welshimeri* isolates; Lane M: 100 bp molecular ladder (ThermoFisher^TM^ Scientific, (EU) Lithuana); Lane N: negative PCR control (no DNA). Figure S4. PCR products of the amplification of *sulI* gene. Lane 1–9: *Listeria* isolates that harbour *sulI* gene (822 bp); lane M: 100 bp molecular weight marker (ThermoFisher^TM^ Scientific, (EU) Lithuana); Lane N: negative PCR control (no DNA). Figure S5. PCR products of the amplification of *tet*A gene. Lane 1–12: *Listeria* isolates that harbour *tet*A gene (201 bp); lane M: 100 bp molecular weight marker (ThermoFisher^TM^ Scientific, (EU) Lithuana); Lane N: negative PCR control (no DNA).
