# Supplementary material for: Multidrug-Resistant Listeria Species Shows Abundance in Environmental Waters of a Key District Municipality in South Africa
Source: Int J Environ Res Public Health. 2021 Jan 8;18(2):481. doi: 10.3390/ijerph18020481 (PMC7826511; doi:10.3390/ijerph18020481)
Supplement: Supplementary file 1 [file ijerph-18-00481-s001.pdf]

<u>Supplementary Figures</u>

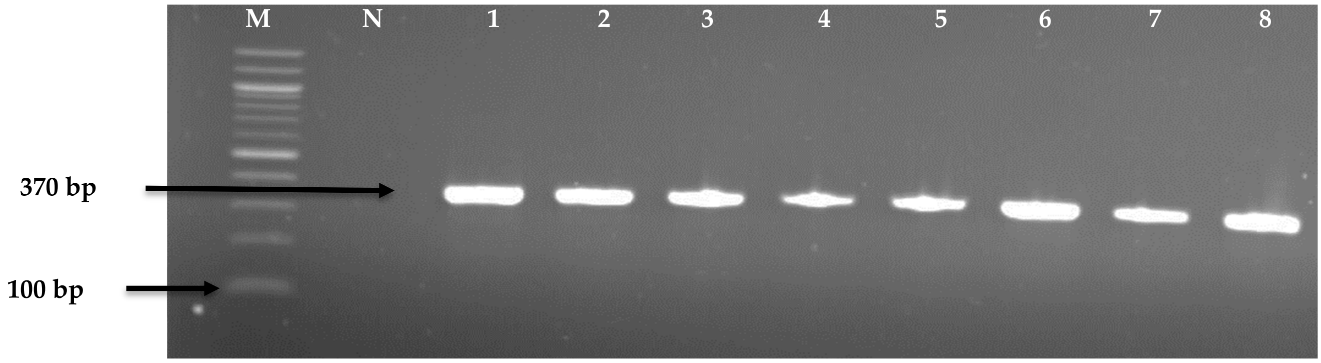

Supplementary Figure S1. PCR products of the amplification of *prs* gene. Lane 1-8: positive *Listeria* spp. isolates; Lane M: 100bp molecular weight marker (ThermoFisher™ Scientific, (EU) Lithuana); Lane N: negative PCR control (no DNA).

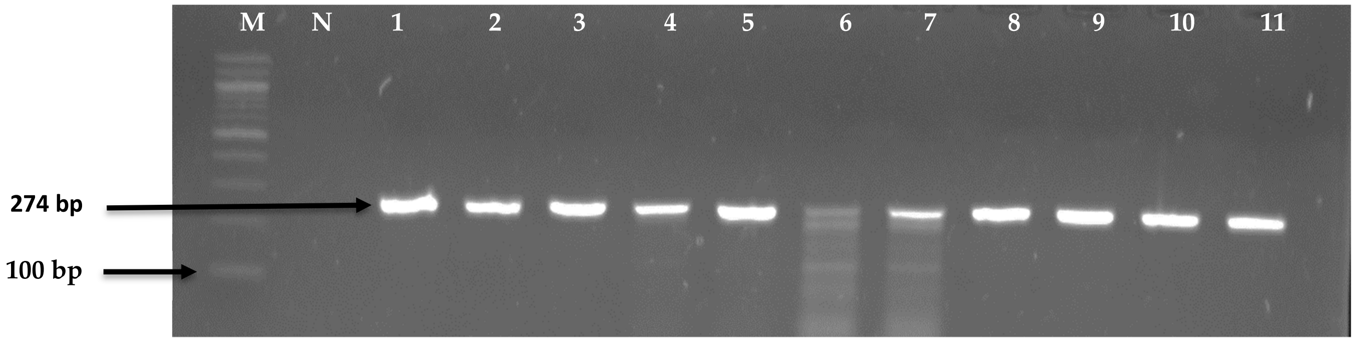

Supplementary Figure S2. PCR products of the amplification of *prf*A gene. Lane 1-11: positive *L. monocytogenes* isolates; Lane M: 100bp molecular weight marker (ThermoFisher™ Scientific, (EU) Lithuana); Lane N: negative PCR control (no DNA).

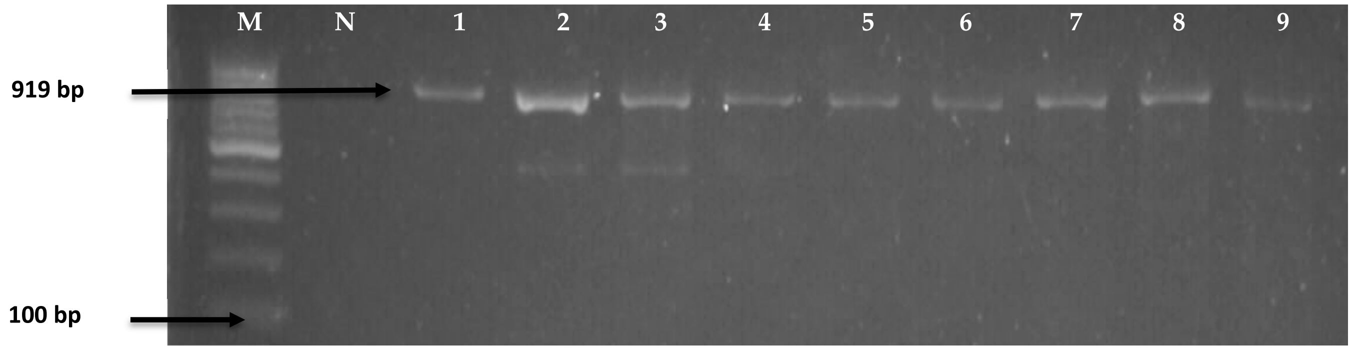

Supplementary Figure S3. PCR products of the amplification of 16S rRNA gene. Lane 1-9: positive *L. welshimeri* isolates; Lane M: 100bp molecular ladder (ThermoFisher™ Scientific, (EU) Lithuana); Lane N: negative PCR control (no DNA).

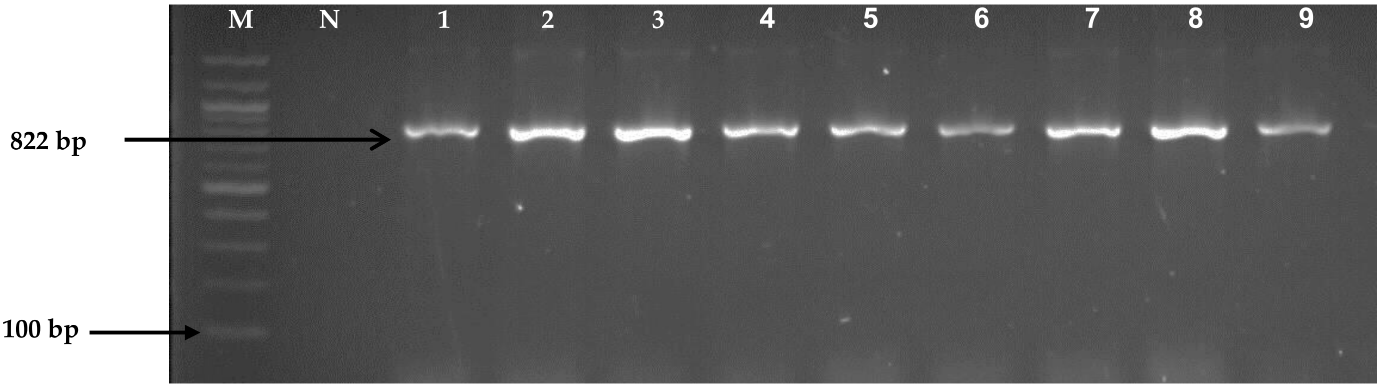

Supplementary Figure S4. PCR products of the amplification of *sulI* gene. Lane 1-9: *Listeria* isolates that harbour *sulI* gene (822 bp); lane M: 100bp molecular weight marker (ThermoFisher™ Scientific, (EU) Lithuana); Lane N: negative PCR control (no DNA).

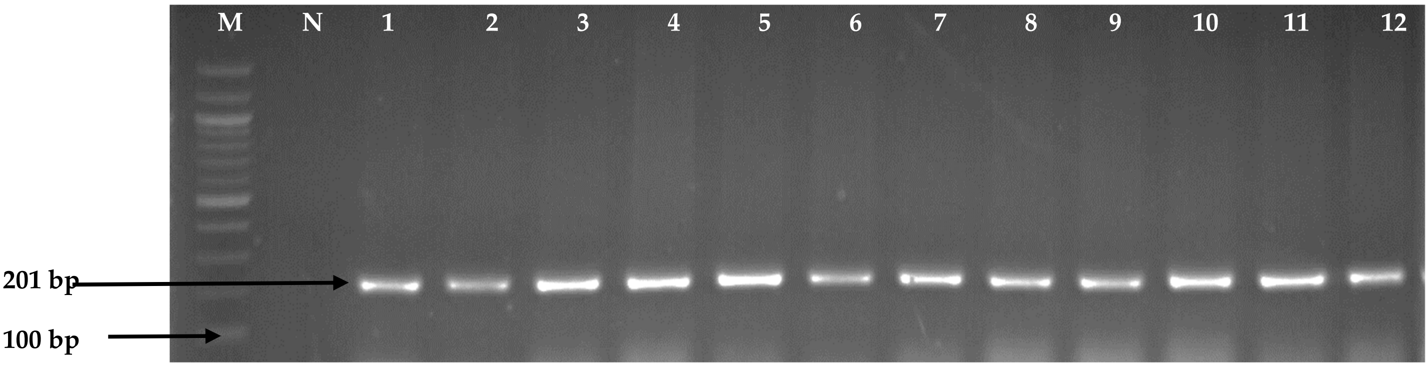

Supplementary Figure S5. PCR products of the amplification of *tet*A gene. Lane 1-12: *Listeria* isolates that harbour *tet*A gene (201 bp); lane M: 100bp molecular weight marker (ThermoFisher™ Scientific, (EU) Lithuana); Lane N: negative PCR control (no DNA).
